# Brain-Wide Resting-State Functional Connectivity Partially Mediates Socioeconomic Disparities in Children’s Cardiometabolic Health

**DOI:** 10.31586/jcn.2025.1143

**Published:** 2025-01-23

**Authors:** Shervin Assari, Hossein Zare, Golnoush Akhlaghipour, Mario F Mendez

**Affiliations:** 1 Department of Internal Medicine, Charles R. Drew University of Medicine and Science, Los Angeles, CA, United States; 2 Department of Family Medicine, Charles R. Drew University of Medicine and Science, Los Angeles, CA, United States; 3 Department of Urban Public Health, Charles R. Drew University of Medicine and Science, Los Angeles, CA, United States; 4 Marginalization-Related Diminished Returns (MDRs) Center, Los Angeles, CA, United States; 5 Department of Health Policy and Management, Johns Hopkins Bloomberg School of Public Health, Baltimore, MD, United States; 6 School of Business, University of Maryland Global Campus (UMGC), Adelphi, United States; 7 Department of Neurology, University of California Los Angeles (UCLA), Los Angeles, CA, USA; 8 Department of Psychiatry & Biobehavioral Sciences, University of California Los Angeles (UCLA), Los Angeles, CA, USA

**Keywords:** Resting-State Functional Connectivity, Socioeconomic Status, Body Mass Index, Blood Pressure, ABCD Study, Factor Analysis, Brain-Wide Analysis, Mediation Analysis, Cardiometabolic Health

## Abstract

**Background:**

Although some neural mechanisms underlying socioeconomic status (SES) disparities are known, the role of brain-wide resting-state functional connectivity in these effects remains less understood.

**Aim:**

This study aims to identify brain-wide resting-state functional connectivity signatures that may mediate the effects of SES on body mass index (BMI) and blood pressure in children, using data from the Adolescent Brain Cognitive Development (ABCD) study.

**Methods:**

Data were drawn from the ABCD study, a large, diverse cohort of children aged 9-10. Pre-processed resting-state functional MRI data were used, and factor analysis was conducted to extract a whole- brain connectivity factor. The first factor, capturing the greatest variance in brain-wide resting-state connectivity, was selected for further analysis in a structural equation model (SEM). This connectivity factor was tested as a potential mediator of the relationship between SES (measured by parental education, family income, and neighborhood characteristics) and two indicators of cardiometabolic health: BMI and systolic blood pressure.

**Results:**

Factor analysis revealed a robust first factor that accounted for a significant proportion of variance in brain-wide resting-state functional connectivity. This factor was significantly associated with SES, indicating that children from lower SES backgrounds exhibited distinct connectivity patterns. Additionally, the factor was linked to both BMI and systolic blood pressure, suggesting its relevance to cardiometabolic health. Mediation analysis showed that this connectivity factor partially mediated the relationship between SES and both BMI and systolic blood pressure.

**Conclusions:**

Brain-wide functional connectivity may be a mediator of SES effects on BMI and blood pressure in children. The first connectivity factor provides a promising neural signature linking SES with cardiometabolic risk. Comprehensive brain- wide approaches to functional connectivity may offer valuable insights into how social determinants of health shape neural and physical development in childhood.

## 1. Introduction

Resting-state functional connectivity has proven valuable for understanding brain function [1–5], but most research in this area has traditionally focused on regions of interest or connectivity within specific brain networks [6–8]. However, recent studies suggest that brain-wide resting-state functional connectivity may yield promising insights [9–11]. Advances in big data analytics now enable researchers to examine neural activity patterns across the entire brain [12–14].

Brain-wide resting-state functional connectivity refers to patterns of neural activity occurring when the brain is at rest, free from specific tasks or external stimuli. These patterns are not random; rather, they reflect intrinsic communication between various brain regions organized into well-established networks crucial for cognitive, emotional, and reward functions. By analyzing connectivity across the whole brain, researchers gain a deeper understanding of large-scale neural organization, showing how the brain as a whole interacts in a coordinated manner at rest. This brain-wide connectivity can then be correlated with social, environmental, or genetic factors, as well as behavioral, emotional, and cognitive outcomes.

It is of particular interest to explore whether brain-wide resting-state functional connectivity can serve as a signature of socioeconomic status (SES) [15]. SES, encompassing factors such as family income, parental education, and neighborhood characteristics, has consistently been linked to disparities in cognitive development and health outcomes. Yet, only recently have researchers begun investigating how these differences manifest in the brain. Studies on resting-state connectivity have identified distinct neural activity patterns associated with SES, particularly in networks related to stress regulation, emotion processing, executive function, and reward. These findings suggest that SES influences the developing brain’s intrinsic architecture, potentially affecting how children respond to environmental stressors and cognitive challenges.

In addition to SES, brain-wide resting-state functional connectivity may also serve as a marker of cardiometabolic health, which is influenced by factors such as emotion regulation and reward processing. Higher body mass index (BMI) has been associated with changes in brain structure, function, and connectivity [16, 17]; however, limited research explores how brain-wide resting-state connectivity reflects these metabolic changes. Even less is known about the neurocognitive correlates of systolic blood pressure, which may be linked to the sympathetic nervous system and the body’s response to stress. Identifying such connectivity signatures could provide critical insights into the neural basis of cardiometabolic outcomes in children.

The significance of studying brain-wide resting-state functional connectivity signatures lies in their potential to elucidate how the social environment impacts a wide range of health, behavioral, and cognitive outcomes [15]. SES-related differences in brain connectivity may influence physiological factors such as BMI and systolic blood pressure in children. Evidence suggests that children from lower SES backgrounds may exhibit altered connectivity in brain regions associated with self-regulation, stress response, and impulse control—factors closely linked to behaviors affecting BMI and cardiovascular health. By identifying neural signatures correlated with both SES and health outcomes, researchers can better understand how the brain mediates the social environment’s impact on physical health markers like BMI and blood pressure.

Using data from the Adolescent Brain Cognitive Development (ABCD) study [18–27], this study aimed to identify a brain-wide resting-state functional connectivity signature that could potentially correlate with both SES and cardiometabolic health in children. It is also hypothesized that this brain-wide resting-state functional connectivity signature may partially mediate the effects of SES on BMI and systolic blood pressure in children.

## 2. Methods

### 2.1. Study Design and Participants

This study utilized data from the Adolescent Brain Cognitive Development (ABCD) study [18–27], a large-scale, longitudinal dataset that follows a diverse cohort of children aged 9-10 across the United States. The ABCD study provides a comprehensive collection of behavioral, demographic, and neuroimaging data, allowing for an in-depth exploration of factors affecting brain and physical development. For this analysis, we used data from the baseline assessment, focusing on a cross-sectional sample to examine resting-state functional connectivity patterns in relation to SES, BMI, and systolic blood pressure. Exclusion criteria included missing data on neuroimaging or cardiometabolic measures and any neurological conditions that could affect resting-state connectivity.

### 2.2. Measures

#### Body Mass Index (BMI) and Systolic Blood Pressure:

Cardiometabolic outcomes included BMI, calculated from height and weight measurements, and systolic blood pressure, measured using a standard sphygmomanometer. Both measures were used as continuous variables in the analysis to capture variability in cardiometabolic health.

#### Socioeconomic Status (SES):

SES was assessed using a composite index derived from family income, parental education level, and neighborhood characteristics. These factors were combined to create a standardized SES score for each participant. Higher scores indicated higher economic status.

### 2.3. Neuroimaging Data Acquisition and Preprocessing

#### MRI Data Acquisition:

Resting-state functional MRI (rs-fMRI) data were collected using a 3T scanner with standardized parameters across study sites. Participants were instructed to keep their eyes open and remain still during the 6-minute scan to ensure consistent resting-state conditions.

#### Preprocessing:

Preprocessing of the rs-fMRI data was conducted using the ABCD pipeline, which included motion correction, slice timing correction, spatial normalization to standard Montreal Neurological Institute (MNI) space, and spatial smoothing with a 6- mm Gaussian kernel. Motion artifacts were minimized by excluding frames with significant head movement (>0.5 mm framewise displacement), and nuisance signals (e.g., white matter, cerebrospinal fluid) were regressed out to reduce noise.

### 2.4. Brain-Wide Resting-State Functional Connectivity Analysis

Connectivity matrices were created by dividing the brain into 268 nodes based on the Shen functional atlas, and calculating Pearson correlations between the time series of each pair of nodes. This resulted in a symmetric connectivity matrix representing brain-wide resting-state functional connectivity for each participant. These matrices were subsequently used for factor analysis.

### 2.5. Factor Analysis

To identify underlying patterns in brain-wide resting-state functional connectivity, we conducted a factor analysis using Stata. The correlation matrices were inputted into a principal components analysis (PCA), with eigenvalues greater than 1 retained. The first factor, capturing the largest proportion of variance in connectivity data, was selected for further analysis. This factor was interpreted as representing a brain-wide signature of resting-state connectivity, potentially reflecting large-scale integration across networks.

### 2.6. Statistical Analysis

We used Stata 18 for data analysis. Mediation analyses were performed using the first factor derived from the factor analysis to test whether brain-wide resting-state connectivity mediated the relationship between SES and cardiometabolic outcomes (BMI and systolic blood pressure). A linear regression model was first used to assess the direct association between SES and the first factor. Then, separate regression models tested the association between the first factor and BMI, as well as systolic blood pressure. The mediation effect was evaluated using SEM in Stata, estimating the indirect effect of SES on cardiometabolic outcomes through the brain-wide resting state functional connectivity. All significance tests were two-tailed, with a threshold of p < 0.05.

### 2.7. Ethical Considerations

The ABCD study protocol was approved by institutional review boards at participating research institutions, and informed consent was obtained from parents or legal guardians of all participants. Data were de-identified to ensure participant privacy and confidentiality, following strict data protection guidelines.

## 3. Results

Table 1 presents the bivariate correlations among the primary study variables. Brain- Wide Resting-State Functional Connectivity was positively correlated with being White (r = 0.15, p < 0.05), household income (r = 0.11, p < 0.05), parental education (r = 0.10, p < 0.05), and marital household status (r = 0.12, p < 0.05). A negative correlation was observed with being Black (r = −0.14, p < 0.05) and BMI (r = −0.13, p < 0.05).

Race/Ethnicity correlations showed that being White was positively correlated with neighborhood income (r = 0.37, p < 0.05), household income (r = 0.43, p < 0.05), and parental education (r = 0.36, p < 0.05), while being Black and Latino were negatively correlated with income (r = −0.32, p < 0.05 and r = −0.20, p < 0.05, respectively), household income (r = −0.37, p < 0.05 and r = −0.22, p < 0.05, respectively), and parental education (r = −0.22, p < 0.05 and r = −0.30, p < 0.05, respectively). Being Black was positively correlated with BMI (r = 0.17, p < 0.05).

Household Income showed positive correlations with married household status (r = 0.55, p < 0.05), parental education (r = 0.62, p < 0.05), and brain-wide resting-state functional connectivity (r = 0.11, p < 0.05), while being negatively correlated with BMI (r = −0.26, p < 0.05) and systolic blood pressure (r = −0.08, p < 0.05). Parental Education was positively associated with neighborhood income (r = 0.38, p < 0.05), married household status (r = 0.35, p < 0.05), and parental employment (r = 0.26, p < 0.05) and negatively correlated with BMI (r = −0.23, p < 0.05). BMI demonstrated a positive correlation with Black race (r = 0.17, p < 0.05) and a negative association with brain-wide resting-state functional connectivity (r = −0.13, p < 0.05), household income (r = −0.26, p < 0.05), and parental education (r = −0.23, p < 0.05). Systolic Blood Pressure had a positive correlation with BMI (r = 0.26, p < 0.05) and showed weaker correlations with other study variables.

Table 2 shows the summary of SEM.

### Associations with BMI:

Brain-wide rsFC was significantly negatively associated with BMI (B = −0.082, SE = 0.010, 95% CI [−0.103, −0.062], p < 0.001), indicating that lower rsFC was associated with higher BMI. Black (B = 0.115, SE = 0.011, 95% CI [0.094, 0.136], p < 0.001) and Latino (B = 0.112, SE = 0.010, 95% CI [0.093, 0.131], p < 0.001) racial backgrounds were positively associated with BMI. Male gender was negatively associated with BMI (B = −0.024, SE = 0.009, 95% CI [−0.042, −0.006], p = 0.008). Age showed a positive association with BMI (B = 0.076, SE = 0.009, 95% CI [0.059, 0.094], p < 0.001). Family income was significantly negatively associated with BMI (B = −0.157, SE = 0.012, 95% CI [−0.181, −0.133], p < 0.001), as was living in a married household (B = −0.050, SE = 0.011, 95% CI [−0.072, −0.027], p < 0.001).

### Associations with Systolic Blood Pressure:

rsFC was negatively associated with systolic blood pressure (B = −0.034, SE = 0.017, 95% CI [−0.067, −0.002], p = 0.040), suggesting that lower connectivity was linked to higher systolic blood pressure. Black racial background was positively associated with systolic blood pressure (B = 0.070, SE = 0.019, 95% CI [0.033, 0.107], p < 0.001).

Male gender was positively associated with systolic blood pressure (B = 0.052, SE = 0.015, 95% CI [0.021, 0.082], p = 0.001), as was age (B = 0.117, SE = 0.015, 95% CI [0.087, 0.148], p < 0.001). Family income was negatively associated with systolic blood pressure (B = −0.049, SE = 0.022, 95% CI [−0.091, −0.006], p = 0.025), though marital status showed no significant association with systolic blood pressure (B = −0.004, SE = 0.020, 95% CI [−0.044, 0.035], p = 0.826).

### Predictors of rsFC:

Family income was marginally associated with rsFC (B = 0.023, SE = 0.014, 95% CI [− 0.004, 0.050], p = 0.099), while marital household status was positively associated with rsFC (B = 0.047, SE = 0.013, 95% CI [0.022, 0.072], p < 0.001). Black (B = −0.143, SE = 0.012, 95% CI [−0.166, −0.119], p < 0.001) and Latino (B = −0.076, SE = 0.011, 95% CI [−0.097, −0.055], p < 0.001) racial backgrounds were significantly negatively associated with rsFC. Male gender was positively associated with rsFC (B = 0.021, SE = 0.010, 95% CI [0.001, 0.041], p = 0.037). Age showed a small but significant negative association with rsFC (B = −0.030, SE = 0.010, 95% CI [−0.049, −0.010], p = 0.004).

## 4. Discussion

This study investigated the role of brain-wide resting-state functional connectivity as a mediator of SES effects on BMI and systolic blood pressure in children. Utilizing data from the ABCD study, we conducted a factor analysis to extract underlying patterns in brain-wide connectivity. The first factor from this analysis, which captured the most variance in resting-state functional connectivity, was examined in relation to SES and cardiometabolic outcomes. Our results indicated that the first factor was significantly associated with SES, suggesting distinct connectivity patterns related to SES background. Furthermore, this connectivity factor was linked to BMI and systolic blood pressure, partially mediating the effects of SES on these health outcomes.

The findings support the hypothesis that brain-wide resting-state functional connectivity can act as a neural signature linking SES [15] to cardiometabolic risk factors. The significant association between the first factor of connectivity and both SES and health outcomes underscores the interconnectedness between social environment and neural development. This study provides evidence that disparities in SES conditions can influence children’s neural functioning, which in turn affects key health markers like BMI and blood pressure. These results align with previous research suggesting that the neural correlates of SES are involved in brain regions associated with self-regulation, stress response, and reward processing—networks that are critical for behaviors related to health and well-being.

Most of the existing literature on resting-state functional connectivity has concentrated on specific brain networks or regions of interest (ROI), often narrowing the analysis to particular areas such as the prefrontal cortex, amygdala, or the default mode network [6–8]. This network-specific or ROI approach has yielded important insights into how targeted brain regions are impacted by various factors, including SES and health outcomes. However, these studies may miss the broader picture by not accounting for the complex, interconnected nature of the brain, where global patterns of connectivity often play a critical role in shaping behavior and health. In contrast, the current study takes a comprehensive, brain-wide approach to examine functional connectivity across the entire brain. This broader perspective allows for the identification of neural patterns that are not confined to isolated networks but instead reflect large-scale, integrative processes. By applying factor analysis to brain-wide connectivity data, our study uncovers connectivity signatures that serve as mediators between SES and cardiometabolic health, providing a more holistic understanding of how social environments influence neural and physical development in children.

Our findings also indicated that Black individuals in the study tend to have higher BMI and systolic blood pressure compared to Whites. In contrast, Latino children showed higher BMI but did not exhibit higher systolic blood pressure compared to their White counterparts. These results highlight important racial and ethnic disparities in health out- comes, with Black children facing elevated risks for both obesity and hypertension, while Latino children, despite having higher BMI, may not still show the same pattern in systolic blood pressure.

### 4.1. Implications

The identification of a brain-wide resting-state connectivity factor that mediates SES effects on BMI and blood pressure has several important implications. First, it emphasizes the utility of a brain-wide approach in understanding the complex interactions between SES factors and health outcomes. Traditional analyses often focus on isolated brain regions or networks, but our results suggest that a comprehensive connectivity analysis can capture the broader neural context that may be relevant for both cognitive and physical development. Additionally, these findings highlight the importance of addressing SES disparities as part of public health strategies, as the neural differences linked to SES may have cascading effects on children’s health trajectories. Targeted interventions that consider both social and biological factors may be particularly effective in mitigating these disparities.

### 4.2. Limitations

There are several limitations to this study that should be acknowledged. First, while the use of a large, diverse sample from the ABCD study enhances the generalizability of our findings, the cross-sectional nature of the data limits our ability to make causal inferences about the relationship between SES, brain connectivity, and health outcomes. Longitudinal studies are necessary to determine the directionality of these effects and whether changes in SES or connectivity over time impact BMI and blood pressure. Additionally, factor analysis provides a data-driven approach to identify underlying patterns in connectivity, but the interpretation of these factors can be challenging. The first factor, while statistically significant, may represent a complex combination of connectivity changes that are not easily linked to specific networks or brain regions.

### 4.3. Future Directions

Future research should focus on validating these findings with longitudinal data to establish the temporal dynamics between SES, brain connectivity, and cardiometabolic outcomes. It would also be valuable to investigate whether targeted interventions aimed at improving social environments or reducing stress can modify these connectivity patterns and, consequently, health outcomes in children. Expanding the analysis to include other health markers, such as mental health or academic performance, could provide a more comprehensive understanding of how SES affects overall well-being through neural mechanisms. Additionally, integrating other advanced neuroimaging techniques and machine learning approaches could refine the identification of brain-wide signatures, enhancing their predictive power for health outcomes.

Future research should aim to address several limitations inherent in this study. Currently, our analysis is limited to resting-state functional connectivity, focusing on an overall youth sample of this specific age range. This approach captures general trends, but does not account for potential heterogeneity within subpopulations, such as differences by sex, ethnicity, or varying levels of SES. Additionally, while the study successfully identifies brain-wide connectivity patterns linked to BMI and systolic blood pressure, it does not examine other important cardiometabolic outcomes or explore associations with broader health indicators like mental health, academic performance, or behavioral traits. Future work should expand this brain-wide methodology to include task-based functional connectivity analyses, different age ranges, and more specific subgroups to identify how diverse factors influence neural connectivity across development. A more detailed exploration of other health outcomes and a breakdown of potential variability within populations could provide a more nuanced understanding of how SES impacts the brain and body, leading to more targeted and effective interventions.

## 5. Conclusions

This study demonstrates that brain-wide resting-state functional connectivity can serve as a mediator of SES effects on BMI and systolic blood pressure in children. The use of factor analysis to extract brain-wide connectivity patterns revealed that SES disparities are reflected in the intrinsic neural architecture of the developing brain, with significant implications for children’s health. Addressing SES-related disparities requires a multifaceted approach, combining efforts to improve social conditions with strategies that consider the biological underpinnings of health outcomes. Our findings underscore the importance of understanding the neural basis of social determinants of health, paving the way for more effective, evidence-based interventions to promote health equity in children.

## Figures and Tables

**Figure 1.**
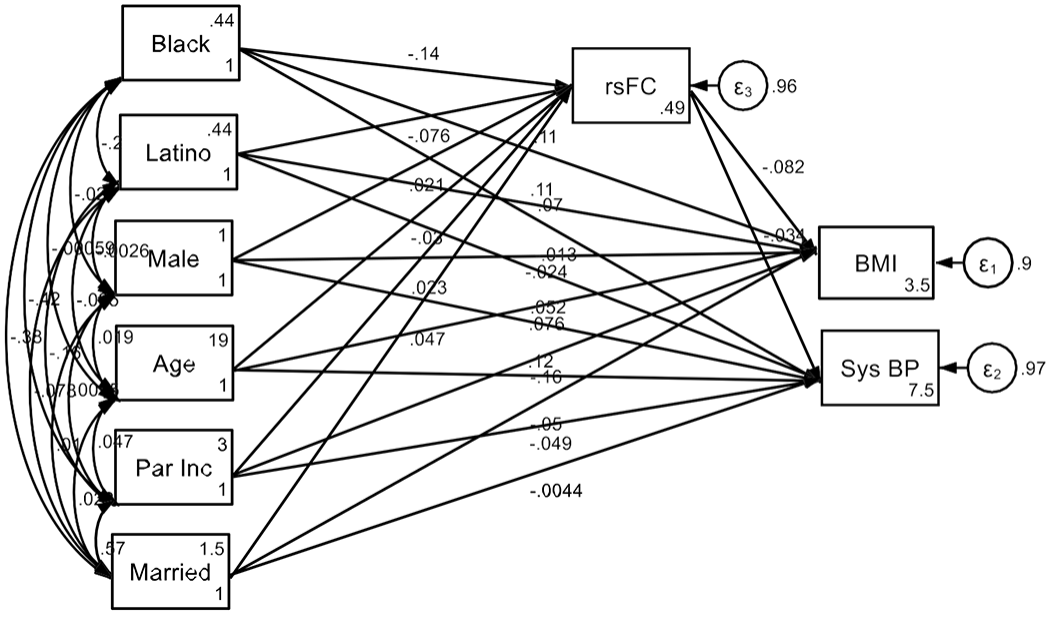
Summary of SEM

**Table 1.** Bivariate correlations between study variables

	1	2	3	4	5	6	7	8	9	10	11	12	13	14	15
1 Brain-Wide resting State Functional Connectivity	1.00														
2 White	0.15	1.00													
3 Black	−0.14	−0.44	1.00												
4 Latino	−0.06	−0.52	−0.21	1.00											
5 Asian	0.03	−0.15	−0.06	−0.07	1.00										
6 Other Race	−0.03	−0.36	−0.14	−0.17	−0.05	1.00									
7 Neighborhood Income / 50000	0.10	0.37	−0.32	−0.20	0.05	0.00	1.00								
8 Household Income	0.11	0.43	−0.37	−0.22	0.06	−0.02	0.46	1.00							
9 Parental Education (Years)	0.10	0.36	−0.22	−0.30	0.10	0.02	0.38	0.62	1.00						
10 Married Household	0.12	0.33	−0.35	−0.11	0.07	−0.02	0.30	0.55	0.35	1.00					
11 Parents Employed	0.00	0.08	−0.04	−0.06	0.01	−0.01	0.13	0.26	0.26	0.04	1.00				
12 Body Mass Index (BMI)	−0.13	−0.23	0.17	0.14	−0.04	0.00	−0.18	−0.26	−0.23	−0.19	−0.03	1.00			
13 Systolic Blood Pressure	−0.05	−0.05	0.10	−0.02	−0.02	0.01	−0.05	−0.08	−0.08	−0.07	0.02	0.26	1.00		
14 Puberty (Any)	−0.02	−0.08	0.09	0.01	0.01	0.01	−0.06	−0.07	−0.05	−0.07	0.00	0.12	0.03	1.00	
15 Male	0.02	0.02	−0.02	0.00	−0.01	0.00	0.01	0.01	0.01	0.01	−0.01	−0.03	0.06	−0.08	1.00

*Numbers larger than 0.01 are statistically significant*.

**Table 2.** Summary of SEM

			B	SE	95%	CI	p
							
rsFC	→	BMI	−0.082	0.010	−0.103	−0.062	< 0.001
Black	→	BMI	0.115	0.011	0.094	0.136	< 0.001
Latino	→	BMI	0.112	0.010	0.093	0.131	< 0.001
Male	→	BMI	−0.024	0.009	−0.042	−0.006	0.008
Age	→	BMI	0.076	0.009	0.059	0.094	< 0.001
Family Income	→	BMI	−0.157	0.012	−0.181	−0.133	< 0.001
Married Household	→	BMI	−0.050	0.011	−0.072	−0.027	< 0.001
Intercept	→	BMI	3.488	0.177	3.141	3.836	< 0.001
							
rsFC	→	Sys BP	−0.034	0.017	−0.067	−0.002	0.040
Black	→	Sys BP	0.070	0.019	0.033	0.107	< 0.001
Latino	→	Sys BP	−0.013	0.017	−0.047	0.020	0.425
Male	→	Sys BP	0.052	0.015	0.021	0.082	0.001
Age	→	Sys BP	0.117	0.015	0.087	0.148	< 0.001
Family Income	→	Sys BP	−0.049	0.022	−0.091	−0.006	0.025
Married Household	→	Sys BP	−0.004	0.020	−0.044	0.035	0.826
Intercept	→	Sys BP	7.544	0.320	6.917	8.171	< 0.001
							
Black	→	rsFC	−0.143	0.012	−0.166	−0.119	< 0.001
Latino	→	rsFC	−0.076	0.011	−0.097	−0.055	< 0.001
Male	→	rsFC	0.021	0.010	0.001	0.041	0.037
Age	→	rsFC	−0.030	0.010	−0.049	−0.010	0.004
Family Income	→	rsFC	0.023	0.014	−0.004	0.050	0.099
Married Household	→	rsFC	0.047	0.013	0.022	0.072	< 0.001
Intercept	→	rsFC	0.486	0.193	0.108	0.864	0.012
